# Circulating Plasma Circular RNAs hsa_circ_0001785 and hsa_circ_0079876 as Promising Noninvasive Diagnostic Biomarkers for Breast Cancer: A Case–Control Study

**DOI:** 10.1155/tbj/8008795

**Published:** 2026-07-05

**Authors:** Amirhossein Sangi Nasab Lahijan, Sepideh Abdollahi, Seyed Rohollah Miri, Ghasem Azizi-Tabesh, Mir Saeed Yekaninejad, Pantea Izadi

**Affiliations:** ^1^ Department of Medical Genetics, School of Medicine, Tehran University of Medical Sciences, Tehran, Iran, tums.ac.ir; ^2^ Cancer Research Center, Cancer Institute of Iran, Tehran University of Medical Sciences, Tehran, Iran, tums.ac.ir; ^3^ Genomic Research Center, Shahid Beheshti University of Medical Sciences, Tehran, Iran, sbmu.ac.ir; ^4^ Department of Epidemiology and Biostatistics, School of Public Health, Tehran University of Medical Sciences, Tehran, Iran, tums.ac.ir

**Keywords:** breast neoplasms, circRNA, noninvasive, plasma

## Abstract

**Background and Aims:**

Breast cancer (BC) is a major health concern for women, with early diagnosis essential for better health outcomes such as survival. While mammography is the gold standard screening tool, it is not without drawbacks. Circular RNAs (circRNAs), a class of noncoding RNAs, characterized by covalently closed loops missing 5′ and 3′ ends, offer stability in body fluids like plasma and are emerging as promising diagnostic biomarkers. Thus, the aim of this study was to investigate the plasma levels of hsa_circ_0001785 and hsa_circ_0079876 in BC patients compared to healthy controls to evaluate their potential for noninvasive BC detection.

**Methods:**

A total of 129 women participated in this study (81 BC patients who underwent surgery and 48 healthy controls based on mammography). The plasma levels of hsa_circ_0001785 and hsa_circ_0079876 were measured using quantitative reverse transcription polymerase chain reaction (qRT‐PCR) following RNA extraction from plasma samples. The diagnostic value was assessed using the receiver operating characteristic (ROC) curve.

**Results:**

A significant increase in the expression of both circRNAs was observed in BC patients compared with healthy controls (*p* value < 0.001). ROC curve analysis demonstrated that an area under the curve (AUC) for hsa_circ_0001785, hsa_circ_0079876, and their combination were 0.76 (sensitivity = 74%, specificity = 79%), 0.98 (sensitivity = 93%, specificity = 92%), and 0.98 (sensitivity = 93%, specificity = 92%), respectively.

**Conclusion:**

Plasma levels of hsa_circ_0001785 and hsa_circ_0079876 are significantly elevated in BC patients, with hsa_circ_0079876 showing superior sensitivity and specificity compared to hsa_circ_0001785. These findings highlight hsa_circ_0079876 as a particularly promising diagnostic potential in a case–control setting for noninvasive BC detection.

## 1. Introduction

Breast cancer (BC) continues to be one of the most widespread cancers among women, hence imposing a significant global health impact. In the last few decades, the incidence of BC has surged by 57.8%, with projections estimating 2.64 million cases globally by 2030 [1]. Early‐stage diagnosis significantly improves treatment outcomes, representing a survival rate for five years exceeding 90%, compared to a mere 20% for patients with distant metastases [2]. Despite continuous advances in BC research field, BC still has a high mortality rate due to delayed diagnosis, tumor heterogeneity, and gaining new mutations during the treatment course, which reduce the effectiveness of treatments and survival rates. Although mammography plays a routine role in early detection, it has several limitations, including potential misdiagnosis (8%–10% false‐positive rate), high costs, and exposure to ionizing radiation [3]. Consequently, there is a critical need for reliable and noninvasive diagnostic tests for BC to facilitate earlier diagnosis and improve treatment outcomes.

Blood‐derived biomarkers, such as circulating tumor DNA (ctDNA), offer a promising alternative as a quick, easy‐to‐accept, and minimally invasive diagnostic method [4]. These markers can be integrated into routine clinical practice, benefiting from their noninvasive nature, which avoids the challenges associated with traditional biopsy procedures. The potential of noninvasive approaches, like liquid biopsy, is further supported by emerging oncogenic panels, which use multiple biomarkers to enhance diagnostic accuracy and guide personalized treatment strategies [5]. These panels are increasingly being validated, offering real‐time tumor profiling and dynamic monitoring of disease progression, making them suitable for widespread clinical implementation. For example, technologies such as next‐generation sequencing (NGS) and digital droplet PCR (ddPCR) allow for precise detection of mutations and amplifications in oncogenes like HER2, MYC, and PIK3CA, providing valuable insights into tumor biology and therapeutic response [6]. Despite numerous attempts, no noninvasive biomarker for the detection of BC has been approved. However, biological fluids like saliva, urine, and plasma contain extracellular noncoding RNAs, which are demonstrating potential as noninvasive and affordable methods for diagnosis [7].

Among noncoding RNAs, including long noncoding RNAs (lncRNAs), microRNAs (miRNAs), and circular RNAs (circRNAs), the last group has shown special promise as cancer diagnostic biomarkers [8]. circRNAs are produced through back splicing and form covalently closed loops without free 3′ or 5′ terminal, increasing their resistance to breakdown caused by exonucleases than linear RNAs in blood or plasma [9]. circRNAs participate in a range of regulatory functions through three main mechanisms: acting as miRNA sponges, binding to proteins, and serving as templates for translation into polypeptides [10]. Given their stability and functional roles, it is plausible that circRNAs dysregulation could contribute to the formation of malignancies [11].

According to the previous investigations, circRNAs named hsa_circ_0001785 and hsa_circ_0079876 are overexpressed in breast tumors [12, 13]. hsa_circ_0001785 is derived from the gene encoding elongator complex protein 3 (ELP3), a member of the RNA polymerase II–associated acetyltransferase elongator enzyme complex [14]. ELP3 expression is notably increased in BC and may promote cancer progression through tRNA modification [15]. The anillin actin–binding protein (ANLN) gene on chromosome 7p14.2 is the source of hsa_circ_0079876 [16]. ANLN, which plays a crucial role in cell cycle regulation, has been linked to aggressive cancer phenotypes and poor prognosis [17]. Although hsa_circ_0001785 and hsa_circ_0079876 have been associated with BC development, their clinical utility as diagnostic biomarkers requires further investigation. Therefore, this study aimed to investigate the biomarker potential of plasma hsa_circ_0001785 and hsa_circ_0079876 in patients with BC.

## 2. Materials and Methods

### 2.1. Study Population

The participants included 81 women who had surgery for suspected BC as cases and 48 healthy women as the control group, recruited from Cancer Institute of Imam Khomeini Hospital in Tehran between October 2022 and December 2023. All participants signed informed consent prior to sample collection. The Tehran University of Medical Sciences Research Ethics Committee approved this study in terms of ethics (IR.TUMS.IKHC.REC.1401.228). Healthy controls were women attending the center for routine BC screening who had negative mammography findings and no clinical or imaging evidence of breast lesions. Individuals with benign breast disease, inflammatory or infectious conditions, autoimmune disorders, endocrine abnormalities, or other systemic diseases were excluded based on a structured questionnaire and medical history review. Suspicion of BC in the case group was triggered by abnormal findings on screening or diagnostic mammography (such as suspicious masses, microcalcifications, or architectural distortion), which led to further evaluation by core needle or excisional biopsy. The suspected BC cases were then confirmed through histopathological evaluation of the breast tissue. Patients’ clinicopathological data such as grade, TNM stage, and immunohistochemistry profile were collected. The selection criteria excluded any participants with prior cancer diagnoses, autoimmune diseases, and inflammatory or endocrine disorders. No preoperative treatments such as chemotherapy or radiotherapy were administered to any participants to avoid confounding effects on gene expression. The blood sample was obtained from each participant and transferred into a tube containing EDTA as an anticoagulant.

### 2.2. Plasma Separation and RNA Extraction

Plasma was separated from whole blood using a two‐step centrifugation protocol at 4°C. Samples were first centrifuged at 1500 rpm for 20 min. The supernatant was then transferred to a new RNase‐free tube and centrifuged again at 5000 rpm for 15 min to further remove residual cellular debris and platelets. Plasma quality was assessed using a Thermo Scientific NanoDrop 2000 by measuring absorbance at 414 nm to detect potential hemolysis. Only nonhemolyzed plasma samples were included in the study and subsequently stored at −80°C. For RNA extraction, 400 microliters (μL) of plasma was treated with the TRIzol reagent (RiboEx LS, GeneAll, Korea) and ethanol precipitation was performed. Next, the RNA pellet was resuspended in 20 μL of water devoid of RNase. Finally, the quality and quantity of the extracted RNAs were measured by a spectrophotometer.

### 2.3. Quantitative‐Reverse Transcription Polymerase Chain Reaction (qRT‐PCR)

The extracted RNA was transformed into complementary DNA (cDNA) using a reverse transcription kit (Smobio, Denmark) by following the manufacturer’s instructions. Random hexamer primers were utilized to create cDNA as circRNAs lack a 3′ poly A tail. circRNA expression levels were determined using a Roche LightCycler 96 System and SYBR Green RealQ Plus 2× MasterMix (Ampliqon, Denmark). The reference gene β‐Actin was used in order to standardize the results. The PCR process consisted of 40 cycles of amplification (95°C for 10 s, 60°C for 15 s, and 72°C for 30 s) after an initial denaturation phase of 30 s at 95°C. Five μL of SYBR Green master mix, 1 μL of each forward and reverse primer (primer concentration: 5 pmol), 1 μL of target cDNA, and 2 μL of sterile water made up the reaction mixture. The specificity of the qRT‐PCR was verified by melting curve analysis, which showed the presence of a single peak. Every reaction was carried out in duplicate. The target circRNAs’ relative expression levels were calculated using the 2^−ΔΔCq^ method. Table 1 lists the primer sequences that were used in this investigation.

**TABLE 1:** Primer sequences used for real‐time PCR.

Target RNA	Forward primer sequence	Reverse primer sequence
hsa_circ_0001785	5′‐GCTGTGATGTCAGGGATATTC‐3′	5′‐TCTCTGGTTCTCACATCTCG‐3′
hsa_circ_0079876	5′‐GCAAGAGGAACAGGAATTAAGC‐3′	5′‐GGTGTGCTACGAGCTGGACT‐3′
β‐Actin	5′‐CCCAGCACAATGAAGATCAAGATCAT‐3′	5′‐ATCTGCTGGAAGGTGGACAGCGA‐3′

### 2.4. Statistical Analysis

GraphPad Prism Version 10.1.0 (316) and SPSS Version 22.0 (IBM Corp., Armonk, NY, USA) were used for statistical analysis. Data are presented as mean ± standard deviation (SD). A *p* value < 0.05 was considered statistically significant. Group comparisons were performed using one‐way ANOVA and two‐tailed Student′s *t*‐tests, as appropriate. Categorical variables were analyzed using the chi‐square test. Receiver operating characteristic (ROC) curve analysis was conducted to evaluate the diagnostic performance of circRNAs, and sensitivity and specificity were determined using Youden’s index. In addition, logistic regression analysis adjusted for age was performed to assess whether the expression levels of the studied circRNAs were independently associated with BC.

## 3. Results

### 3.1. Patient Characteristics

Table 2 provides an overview of the study participants’ clinical characteristics. A total of 81 BC patients with invasive ductal carcinoma (IDC, *n* = 51), ductal carcinoma in situ (DCIS, *n* = 29), and lobular carcinoma in situ (LCIS, *n* = 1) were enrolled in this study (mean age: 41.79 ± 6.77 years). The control group included 48 healthy women with mean age of 53.21 ± 11.66 years.

**TABLE 2:** Study participants’ clinical and demographic features.

Characteristic	Patients (*n* = 81) (%)
Mean age (years)	41.79 ± 6.77

*HER2 status*	
Positive	40.7
Negative	59.3

*ER status*	
Positive	72.8
Negative	27.2

*PR status*	
Positive	70.4
Negative	29.6

*Cancer type*	
IDC	63
DCIS	35.8
LCIS	1.2

*TNM stage*	
Stage 0‐I	19.8
Stage II	51.9
Stage III	16
Stage IV	12.3

*Histological grade*	
Low	30.9
High	69.1

*Note:* HER2: human epidermal growth factor receptor 2.

Abbreviations: DCIS = ductal carcinoma in situ, ER = estrogen receptor, IDS = invasive ductal carcinoma, LCIS = lobular carcinoma in situ, PR = progesterone receptor.

### 3.2. Expression Levels of Plasma circRNAs

**FIGURE 1:**
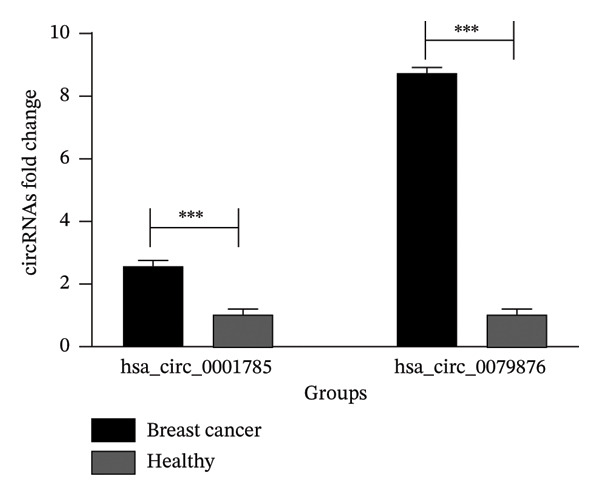
Comparison of plasma expression levels of hsa_circ_0001785 and hsa_circ_0079876 in breast cancer patients relative to healthy controls (^∗∗∗^ *p* value < 0.001).

Using qRT‐PCR, the relative levels of hsa_circ_0001785 and hsa_circ_0079876 in plasma samples were determined. The findings revealed that, in comparison to controls, BC patients had significantly higher plasma levels of both circRNAs (*p* value < 0.001) (Figure 1).

### 3.3. Association of circRNA Expression Levels With Clinical Characteristics

The expression levels of hsa_circ_0001785 and hsa_circ_0079876 did not show significant association with clinicopathological characteristics, including age, BC subtype, histological grade, TNM stage, lymph node invasion, hormone receptors (ER and PR), and HER2 status (Table 3).

**TABLE 3:** Association between clinicopathological characteristics and plasma levels of hsa_circ_0001785 and hsa_circ_0079876.

Variables	Subclass	*N*	Mean ± SD (hsa_circ_0001785)	*p*‐value (hsa_circ_0001785)	Mean ± SD (hsa_circ_0079876)	*p* value (hsa_circ_0079876)
Age	< 50	35	1.87 ± 1.48	0.524	−5.05 ± 1.13	0.146
	≥ 50	46	1.67 ± 1.32		−5.51 ± 1.57	

Breast cancer type	IDC	51	1.83 ± 1.32	0.716	−5.25 ± 1.25	0.488
	DCIS	29	1.61 ± 1.52		−5.36 ± 1.67	
	LCIS	1	2.40		−6.95	

Histological grade	Low grade	25	1.59 ± 1.34	0.453	−5.31 ± 1.78	0.983
	High grade	56	1.84 ± 1.41		−5.32 ± 1.22	

TNM stage	I	16	2.10 ± 1.63	0.226	−5.00 ± 1.88	0.492
	II	42	1.48 ± 1.33		−5.52 ± 1.21	
	III	13	1.80 ± 1.44		−5.36 ± 1.53	
	IV	10	2.33 ± 0.90		−4.92 ± 1.21	

Lymph node invasion	Yes	40	1.67 ± 1.55	0.553	−5.26 ± 1.27	0.751
	No	41	1.85 ± 1.22		−5.36 ± 1.55	

ER status	Positive	59	1.78 ± 1.42	0.841	−5.31 ± 1.48	0.998
	Negative	22	1.71 ± 1.31		−5.32 ± 1.23	

PR status	Positive	57	1.67 ± 1.44	0.366	−5.33 ± 1.48	0.868
	Negative	24	1.98 ± 1.26		−5.27 ± 1.26	

HER2 status	Positive	33	1.75 ± 1.39	0.976	−5.28 ± 1.46	0.849
	Negative	48	1.76 ± 1.40		−5.34 ± 1.39	

*Note:* HER2: human epidermal growth factor receptor 2.

Abbreviations: DCIS = ductal carcinoma in situ, ER = estrogen receptor, IDC = invasive ductal carcinoma, LCIS = lobular carcinoma in situ, PR = progesterone receptor.

### 3.4. Diagnostic Utility

**FIGURE 2:**
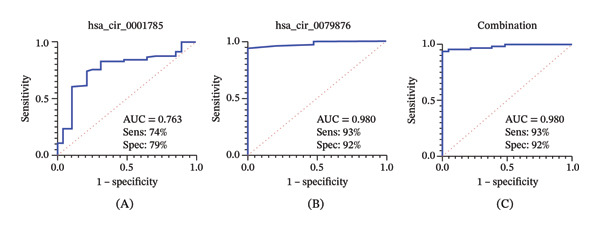
The results of receiver operating characteristic (ROC) curve analysis for the diagnostic value of hsa_circ_0001785 (A) and hsa_circ_0079876 (B) and the area under the curve (AUC) 0.76 with 95% CI (0.6762–0.8505) and 0.98 with 95% CI (0.9598–1.000), respectively. The combination of hsa_circ_0001785 and hsa_circ_0079876 (C) in the plasma of breast cancer patients, which was reported as 0.98.

ROC curve analysis has been employed to determine the diagnostic potential of hsa_circ_0001785 and hsa_circ_0079876. The area under the curve (AUC) for hsa_circ_0001785 was 0.76 (95% confidence interval [CI] 0.676–0.85), accompanied by 0.74 for sensitivity and 0.79 for specificity. Furthermore, an AUC of 0.98 (95% CI 0.959–1.0), 0.92 for specificity, and 0.93 for sensitivity were reported for hsa_circ_0079876. In addition, the combined diagnostic power of hsa_circ_0079876 and hsa_circ_0001785 was equal to that of hsa_circ_0079876 alone (Figure 2).

### 3.5. Age‐Adjusted Logistic Regression Analysis

To evaluate whether the association between circRNA expression levels and BC was independent of age, logistic regression analysis adjusted for age was performed. The results showed that both circRNAs remained significantly associated with BC after age adjustment. Increased expression of hsa_circ_0001785 was independently associated with BC (OR = 1.71, 95% CI: 1.28–2.27, *p* < 0.001). Also, the expression of hsa_circ_0079876 remained independently associated with BC after adjustment for age (OR = 1.96, 95% CI: 1.47–2.63, *p* < 0.001).

## 4. Discussion

The present study evaluated the plasma level of hsa_circ_0001785 and hsa_circ_0079876 in patients with BC in comparison to the normal women. The age distribution of the patients was within the early fifth decade of life, which is consistent with previous epidemiological report indicating that BC in Iranian women tends to occur approximately a decade earlier than in many western populations [18]. The pathological features of the studied patients were also representative of the known distribution of BC subtypes, with IDC being the predominant histological type and a high proportion of tumors showing ER and PR positivity, which reflects the most common molecular phenotype observed in BC (Table 2).

Our findings revealed a significant increase in plasma hsa_circ_0001785 levels in BC patients compared with non‐BC women, with an AUC of 0.76 (sensitivity = 74%, specificity = 79%), indicating a potential diagnostic value. These findings are consistent with a previous study that reported the upregulation of hsa_circ_0001785 in the plasma and tissue of BC patients, with a similar AUC of 0.77 [12]. Interestingly, although hsa_circ_0001785 appears to be elevated in plasma and tissue in previous investigations [12, 19], it has reported reduced expression in BC cell lines [19]. This apparent discrepancy may reflect differences between intracellular expression and circulating release of the mentioned circRNA. One possible explanation is that circRNAs may be selectively exported from tumor cells into the circulation, rather than directly reflecting their intracellular abundance. In addition, circulating circRNA levels may also be influenced by tumor–microenvironment interactions in vivo which is absent in cell line study. Mechanistically, hsa_circ_0001785 has been shown to act as a sponge for miR‐942, leading to the overexpression of its target gene, SOCS3 (suppressor of cytokine signaling 3), thereby reducing the invasion, migration, and proliferation of BC cells [19]. This finding could explain the low level of hsa_circ_0001785 expression in BC cells. However, further research is needed to understand the differential expression of hsa_circ_0001785 in BC cells and plasma.

This study also demonstrated an elevated levels of plasma hsa_circ_0079876 in BC patients compared with non‐BC women, with an AUC of 0.98 (sensitivity = 93%, specificity = 92%). These results corroborate a previous study that reported the overexpression of plasma hsa_circ_0079876 in BC patients [13]. This is also consistent with previous findings, which indicates the upregulation of this circRNA in BC tissue [13]. The high sensitivity and specificity of hsa_circ_0079876 suggest a promising diagnostic potential for BC.

When comparing two evaluated circRNAs in the current study with established serum and liquid biopsy markers, the advantages of plasma circRNAs become evident. Conventional serum biomarkers such as CA15 3 and CEA have repeatedly been reported to exhibit poor sensitivity and specificity, particularly for early detection and minimal residual disease, limiting their value for screening or early diagnosis [20]. In contrast, liquid biopsy–based analytes, including circulating miRNA panels (with reported sensitivity > 85% and specificity > 90%) and multiomic ctDNA/cfDNA assays, have shown improved diagnostic efficiency compared with traditional serum tests [21]. Within this context, the studied plasma circRNA, hsa_circ_0079876, demonstrated strong diagnostic performance, achieving 93% sensitivity and 92% specificity, comparable to or exceeding that of other emerging noninvasive biomarkers. These findings highlight the translational potential of circRNAs, particularly hsa_circ_0079876, as a promising noninvasive biomarker for BC detection in future.

In the present study, the expression levels of hsa_circ_0001785 and hsa_circ_0079876 were not significantly associated with clinicopathological factors such as age, tumor stage, or hormone receptor status (ER, PR, and HER2) (Table 3). It is another advantage of these circRNAs, which shows that their diagnostic performance is not restricted to a specific clinical subgroup of patients. Notably, the age‐independent expression pattern observed in our patients is consistent with previous reports [12, 13], further supporting the stability of these circRNAs across different demographic groups. Furthermore, additional logistic regression analysis adjusted for age demonstrated that both hsa_circ_0001785 and hsa_circ_0079876 remained significantly associated with BC, indicating that their diagnostic performance was not confounded by the age difference between the case and control groups.

Although hsa_circ_0079876 showed superior diagnostic performance, our analysis suggested that hsa_circ_0001785 and hsa_circ_0079876 contribute largely independent information. Thus, the combination of both biomarkers was evaluated for diagnostic modeling. Nevertheless, combining the two circRNAs did not significantly improve diagnostic accuracy beyond that achieved by hsa_circ_0079876 alone. Therefore, given the added cost and complexity of measuring both mentioned circRNAs, hsa_circ_0079876 alone may represent a more practical option for BC detection. Further studies with larger cohorts are needed to validate these findings and assess the potential of combined versus individual circRNA models in different clinical settings. As a limitation, our study did not investigate the potential role of these circRNAs in BC recurrence, which may be an interesting area for future research. It must be considered that our evaluation was at a single time point and the other potential roles of these circRNAs in treatment monitoring or minimal residual disease assessment need further study. Future prospective studies with serial sampling and longitudinal follow‐up, in addition to independent validation in larger cohorts, are needed to clarify the temporal dynamics and clinical utility of these biomarkers.

## 5. Conclusion

In conclusion, our findings suggest that hsa_circ_0001785 and, particularly, hsa_circ_0079876—with their elevated plasma levels—are promising biomarkers with diagnostic potential for BC in clinical practice. Given the current lack of reliable noninvasive biomarkers and the unmet need for early detection, these circRNAs may contribute to improving diagnostic precision. Nevertheless, the biological mechanisms underlying their release into circulation and their physiological and pathological relevance in breast tissue remain unclear. Importantly, to establish their true clinical utility, future research should incorporate longitudinal monitoring and repeated measurements in the same patients. Such study designs are essential for determining whether these circRNAs can consistently track disease dynamics, support early relapse detection, and serve as stable biomarkers over time.

## Author Contributions

Amirhossein Sangi Nasab Lahijan conducted the experiments and wrote the manuscript. Sepideh Abdollahi contributed to laboratory setup and manuscript writing. Seyed Rohollah Miri provided patients’ diagnosis and samples. Ghasem Azizi‐Tabesh contributed in assay design. Mir Saeed Yekaninejad performed statistical analysis. Pantea Izadi supervised the project, interpreted data, and revised the manuscript. The corresponding author had full access to all of the data in this study and takes complete responsibility for the integrity of the data and the accuracy of the data analysis.

## Funding

This study was a part of a M.Sc. Thesis supported by Tehran University of Medical Sciences (Tehran, Iran; grant no: 62989).

## Disclosure

Dr. Pantea Izadi affirms that this manuscript is an honest, accurate, and transparent account of the study being reported; that no important aspects of the study have been omitted; and that any discrepancies from the study as planned have been explained. All authors have read and approved the final version of the manuscript.

## Ethics Statement

The Tehran University of Medical Sciences Research Ethics Committee approved this study in terms of ethics (IR.TUMS.IKHC.REC.1401.228).

## Conflicts of Interest

The authors declare no conflicts of interest.

## Data Availability

The original data presented in the study are included within the article; further inquiries can be directed to the corresponding author.
